# High Performance Pd/4H-SiC Epitaxial Schottky Barrier Radiation Detectors for Harsh Environment Applications

**DOI:** 10.3390/mi14081532

**Published:** 2023-07-30

**Authors:** Krishna C. Mandal, Sandeep K. Chaudhuri, Ritwik Nag

**Affiliations:** Department of Electrical Engineering, University of South Carolina, Columbia, SC 29208, USA

**Keywords:** 4H-SiC, deep level transient spectroscopy (DLTS), defects in semiconductors, epitaxial layer, radiation detection, Schottky barrier diode

## Abstract

Although many refractory metals have been investigated as the choice of contact metal in 4H-SiC devices, palladium (Pd) as a Schottky barrier contact for 4H-SiC radiation detectors for harsh environment applications has not been investigated adequately. Pd is a refractory metal with high material weight-to-thickness ratio and a work function as high as nickel, one of the conventional metal contacts for high performing 4H-SiC Schottky barrier detectors (SBDs). In this article, Pd/4H-SiC epitaxial SBDs have been demonstrated for the first time as a superior self-biased (0 V applied bias) radiation detector when compared to benchmark Ni/4H-SiC SBDs. The Pd/4H-SiC SBD radiation detectors showed a very high energy resolution of 1.9% and 0.49% under self- and optimized bias, respectively, for 5486 keV alpha particles. The SBDs demonstrated a built-in voltage (*V_bi_*) of 2.03 V and a hole diffusion length (*L_d_*) of 30.8 µm. Such high *V_bi_* and *L_d_* led to an excellent charge collection efficiency of 76% in the self-biased mode. Capacitance mode deep level transient spectroscopy (DLTS) results revealed that the “lifetime-killer” Z_1/2_ trap centers were present in the 4H-SiC epilayer. Another deep level trap was located at 1.09 eV below the conduction band minimum and resembles the EH5 trap with a concentration of 1.98 × 10^11^ cm^−3^ and capture cross-section 1.7 × 10^−17^ cm^−2^; however, the detector performance was found to be limited by charge trapping in the Z_1/2_ center. The results presented in this article revealed the unexplored potential of a wide bandgap semiconductor, SiC, as high-efficiency self-biased radiation detectors. Such high performance self-biased radiation detectors are poised to address the longstanding problem of designing self-powered sensor devices for harsh environment applications e.g., advanced nuclear reactors and deep space missions.

## 1. Introduction

With a wide bandgap of ≈3.27 eV at 300 K, 4H-SiC, a polytype of silicon carbide, offers very low leakage current as a rectifying device even at high reverse bias resulting in extremely low thermal noise [1,2,3,4,5]. The dielectric constant of 4H-SiC (9.7) is much lower when compared to 11.9 and 16.0 in Si and Ge, respectively, which helps to lower the detector capacitance for a given detector active volume. The lower detector capacitance in turn helps to achieve higher charge collection efficiency and lower the white series noise [6]. Epitaxially grown 4H-SiC layers exhibit excellent electron transport properties, including high drift mobility (≈1000 cm^2^/V/s), which allows faster charge collection and hence provides fast response time. 4H-SiC epilayer devices also demonstrate high breakdown electric field (3 × 10^6^ V/cm), a saturation electron drift velocity (2 × 10^7^ cm/s) twice that of silicon, and a relatively low electron-hole pair (ehp) generation energy of 7.28 eV. Lower ehp generation energy implies a large Fano factor and thereby a high energy resolution [5,7,8]. A high sublimation point of 2857 °C and thermal conductivity of 280 W/m/K allows 4H-SiC devices and ICs to operate at temperatures as high as 800 °C [9]. A high displacement threshold of Si (35 eV) and C (22 eV) in 4H-SiC makes it difficult for incident radiations to displace any atom from its lattice point, making it radiation hard and hence suitable for applications in environments with high background radiation [10,11]. Due to the above-mentioned superior electronic properties along with its physical ruggedness, 4H-SiC has been demonstrated to be compatible with harsh environment applications such as in nuclear reactor cores, laser plasma, space missions, and high energy astrophysics [12,13,14].

Despite the promising aforementioned properties, a wide variety of microscopic and macroscopic crystal defects exist in the state-of-the-art 4H-SiC wafers which deteriorate the device performance [15,16]. In particular, micropipe defects in 4H-SiC single crystals, which are basically hollow-core threading screw dislocations, are the most potential crystal defects that deteriorate the device performance [17,18]. As a result, over the last decade, much effort has been put into reducing the micropipe defect concentration. Nowadays, 4H-SiC epitaxial layers are regularly grown with micropipe defect density < 1 cm^−2^ [19]. Microscopic trap centers, such as deep levels related to point defects, trap charge carriers, thereby adversely affecting the charge collection and detector performance [20,21]. Semiconductor devices with lower defect concentrations have a higher mobility-lifetime (*μτ*) product, a figure-of-merit for charge transport properties, and exhibit higher energy resolution. Among the best detectors, the concentration of the point defects is reported to be below 10^12^ cm^−3^ [22,23,24]. Among various reported point defects in 4H-SiC, the most significant is the Z_1/2_ “electron lifetime killing” trap center, with an activation energy between 0.6–0.7 eV [25,26].

The metal-4H-SiC Schottky barrier diode forms the most simplistic and effective detector structure, where the thin metal on the semiconductor surface forms a rectifying contact to achieve extremely low leakage currents when reverse biased. The metal contact also acts as a semitransparent window to most incident radiations. Due to its high work function of ≈5.22 eV, Nickel (Ni) is widely used to realize Schottky contact on n-type 4H-SiC with high barrier heights. Ni contacts also facilitate achieving low contact resistivity and resilience to thermal fluctuations [27]. Palladium (Pd) is yet another refractory contact metal with a work function as high as Ni, appropriate for achieving high barrier heights in 4H-SiC. Pd is a metal contact of choice for electronic devices due to its superior material weight-to-thickness ratio [28]. Although Pd/4H-SiC Schottky barrier detectors (SBDs) have been demonstrated as UV detectors, their response to charge particle detection has not been explored fully yet.

In this article, we fabricated Pd/n-4H-SiC SBDs on a 20-μm thick n-type 4H-SiC high-quality epitaxial layer and evaluated their detection properties when exposed to charged particles. The detection properties have been correlated with the Schottky junction parameters derived from electrical measurements and the charge trapping effects in the crystal defects derived from capacitance mode deep level transient spectrometer. The prospect of the fabricated Pd/n-4H-SiC SBD has been assessed for high resolution radiation detection.

## 2. Materials and Methods

**Figure 1:**
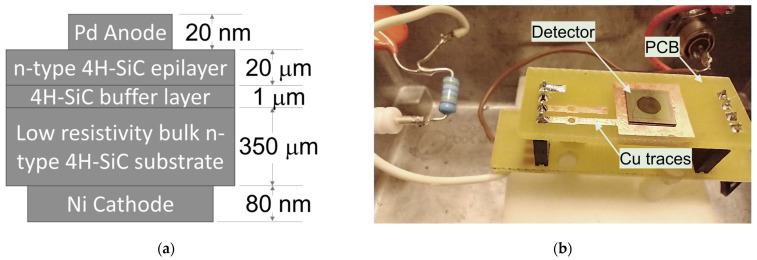
(**a**) Schematic (not to scale) of a Pd/4H-SiC SBD fabricated on a 20 μm thick n-type 4H-SiC epilayer; (**b**) A Pd/4H-SiC SBD detector mounted on a test PCB. The top contact (circular spot) is the Schottky contact and used as a detector window.

N-type 4H-SiC epilayers with an average thickness of 20 µm were grown on the (100) plane of a 100 mm diameter heavily doped n-type 4H-SiC substrate 4° off-cut towards the [11$\bar{2}$0] crystal direction using chemical vapor deposition (CVD). The epitaxial wafer was diced into square specimens with an edge length of 8 mm followed by an RCA cleaning. Ohmic back contacts were obtained by depositing 80 nm thick square (6 mm × 6 mm) Ni using a benchtop Quorum Q150T DC sputter coater. Circular Pd contacts with thickness 20 nm and area ≈11 mm^2^ were sputter coated on the epilayer side to obtain the Schottky junction. The cross-sectional view of the wafer structure and a photograph of a detector used in this study is shown in Figure 1a,b, respectively.

The electrical characterization of the detector was carried out at room temperature under dark ambience through current-voltage and capacitance-voltage measurements using a Keithley 237 source-measure unit and a high frequency (1 MHz) Sula Technologies DDS-12 capacitance meter, respectively. The detectors were mounted on custom-made PCBs installed inside an electronic test-box shielded from electromagnetic interference (EMI). Defect levels were analyzed using a Sula Technologies DDS-12 deep level transient spectrometer (DLTS). The detectors were mounted on a Cu holder installed inside a Janis VPF-800 liquid nitrogen cooled cryostat controlled by a Lakeshore LS335 temperature controller. The DLTS system comprises a pulse generator, a 1 MHz oscillator, a correlator module, a femto-Farad resolution capacitance meter, and a preamplifier module. The DDS-12 allows fully automated DLTS scans in the temperature range 80–800 K controlled by a data acquisition program coded in LabVIEW platform. The detectors were pulsed to 0 V from a steady state reverse bias, $V_{p}$ set at −9 V with a pulse width of 2 msec during which the depletion width collapses and fills the majority carrier (electron) trap centers. Following the pulsing, the bias returned to the steady state immediately, while the trapped electrons gradually detrapped to the conduction band. The relaxation period depended on the rate window setting and varied between 50 and 1000 msec. The DDS-12 system allows one to select twelve rate windows. The time positions of the rate windows (∆τ) are set by initial delays $t_{d}$, ranging from 0.02 msec to 100 msec, measured from the end of the trap-filling pulse. The rate window and the initial delay are related as below.

(1) $$\;∆\tau =4.3{\;\times \;t}_{d}$$

The 4H-SiC detectors were mounted inside an EMI shielded test box for the pulse height spectroscopy. The test box was constantly evacuated during the measurement using a mechanical pump to maintain a vacuum of <1 × 10^−4^ Torr that minimizes the scattering of alpha particles with air molecules. A 0.9 μCi ^241^Am alpha particle source was placed 1.5 cm above the Pd window of the detector. The broad window alpha source was used to ensure uniform illumination of the whole detector window. A Cremat CR110 charge sensitive preamplifier was used to collect the detector signals and an Ortec 671 spectroscopy amplifier was used to filter and amplify the preamplifier output. A Canberra Multiport II ADC/MCA unit controlled by a Genie 2000 data acquisition program was used to acquire the pulse height spectra (PHS).

An absolute calibration method was adopted to correlate the MCA channel numbers to the energy of the detected radiation [29]. A precision pulser generating waveforms similar to detector output signals was connected to the preamplifier input through a feedthrough capacitor with capacitance $C_{t}$. The amplitudes of the charge pulses ($V_{pulser}$, mV) from the capacitor were converted to energy equivalent from a 4H-SiC detector ($E_{pulser}$ in keV) using Equation (2) below:

(2) $$E_{pulser}=\frac{V_{pulser}\times \varepsilon \times C_{t}}{1.6\times 10^{-19}}$$

In the above equation ε is the ehp generation energy (7.3 eV for 4H-SiC). A set of PHS was acquired for different $V_{pulser}$s by adjusting the amplitudes of the pulser output. The corresponding peak position and the energy were plotted in a graph to obtain a correlation between radiation energy and the peak position in the MCA.

The charge collection efficiency (CCE), η, was calculated as the ratio of energy read by the detector ($E_{det}$) to the energy of the incident particles (~5486 keV) when the detector was illuminated by the alpha source. $E_{det}$ was derived from the corresponding alpha peak centroid in the PHS.

The detector energy resolution was calculated as the full width at half maximum (FWHM), ∆E, of the alpha peak in the PHS. The energy resolution has also been expressed as percentage resolution ∆E% calculated as below:

(3) $$∆E\%=\frac{∆E}{E_{det}(keV)}\times 100.$$

## 3. Results

### 3.1. Electrical Characterization

**Figure 2:**
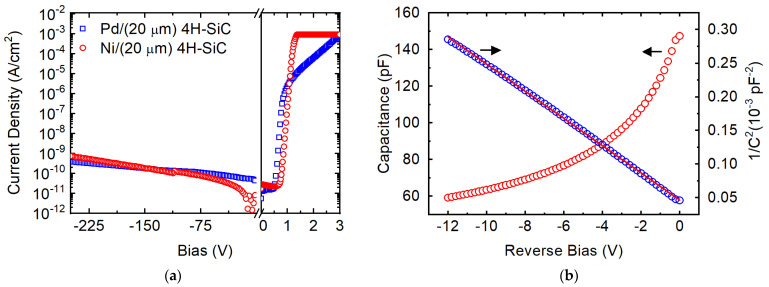
Variation of current density *J* (**a**) and capacitance *C* (**b**) as a function of bias voltage *V* obtained in a Pd/4H-SiC epitaxial SBD. (The solid straight line in (**b**) is the linear fit to the 1/C^2^ vs V plot). The *J-V* plot for a Ni/4H-SiC SBD has also been plotted for comparison.

The current density-voltage (*J-V)* characteristic in Figure 2a shows the variation of leakage current density (*J*) as a function of the detector bias *V*. The bias has been applied to the anode (Schottky) Pd contact and measured (magnitude and polarity) with respect to the cathode (Ohmic) Ni contact kept at the ground potential. The leakage current density was calculated as the ratio of leakage current to the contact area (0.01 cm^2^). A dark current density of 0.4 nA/cm^2^ at −250 V was observed, which is lower than that observed in a state-of-the-art benchmark Ni/4H-SiC SBD. Figure 2a also compares the *J-V* characteristic obtained from the benchmark SBD which exhibited a leakage current density of 0.7 nA/cm^2^ at −250 V. The benchmark detector is among the 4H-SiC detectors that has demonstrated record high energy resolution for 5486 keV alpha particles [6]. Leakage current is an important device parameter as the white parallel noise increases with device leakage current affecting the energy resolution of the detector adversely [30]. With such low leakage currents observed, the Pd/4H-SiC is poised to exhibit very high energy resolution.

The intensity of reverse leakage current in an SBD depends on the Schottky barrier height (SBH). The SBH is usually determined by fitting the experimentally obtained forward bias *J-V* characteristics to a thermionic emission model given by [31,32],

(4) $$J=A^{*}T^{2}\exp\left(-\frac{q\Phi_{B}}{k_{B}T}\right)\left[\exp\left(\frac{qV}{nk_{B}T}\right)-1\right].$$

In the above equation, $A^{*}$ is the effective Richardson constant (146 A-cm^−2^K^−2^ for 4H-SiC), q is the electronic charge, $k_{B}$ is the Boltzmann constant, and T is the absolute temperature. From the fitting, the diode ideality factor n and the SBH $\Phi_{B}$ were calculated to be 1.1 and 1.5 eV, respectively. An ideality factor greater than one suggests that the barrier height is not homogeneously distributed across the entire contact area and there exist regions on the surface with lower barrier heights through which a fraction of the current flows [33]. The calculated barrier height of 1.5 eV is thus the minimum barrier height dominated by the low Schottky barrier height locations. The benchmark Ni/4H-SiC SBD showed similar junction properties with an ideality factor of 1.2 and a Schottky barrier height of 1.6 eV [1,4,6].

The average barrier height for the entire diode area can be determined indirectly from the capacitance-voltage (*C-V*) characteristics [34]. Figure 2b shows the *C-V* characteristics measured for the Pd/4H-SiC SBD along with the Mott–Schottky (*A*^2^/*C*^2^ vs. *V*) plot where *A* is the contact area. From the slope (m) of the linear fit of *A*^2^/*C*^2^ vs. *V* plot and Equation (5) below, the effective doping concentration, $N_{eff}$, in the detector’s active region was calculated to be 1.63 × 10^14^ cm^−3^.

(5) $$N_{eff}=\frac{2}{qA^{2}\varepsilon_{0}\varepsilon_{r}m}$$

where, $\varepsilon_{r}$ is the dielectric constant of 4H-SiC (9.66) and $\varepsilon_{0}$ is the permittivity of vacuum. A lower value of $N_{eff}$ ensures that for a given detector volume the full depletion width may be achieved for a smaller reverse bias. Considering the above-calculated $N_{eff}$, the 20 μm thick epilayer would be depleted fully at a reverse bias as low as −60 V. The penetration depth of 5486 keV alpha particles in 4H-SiC is ≈18 μm, which ensures that, when fully depleted, all the incident alpha particles are stopped within the depletion width, a criteria required to achieve optimum charge collection efficiency and energy resolution.

The intercept of the Mott–Schottky plot on the voltage axis gives the built-in potential *V_bi_*, which was calculated to be 2.03 V. The *V_bi_* for the benchmark Ni/4H-SiC SBD has been reported to be 2.1 V. Such high built-in potential is crucial to obtaining high charge collection efficiency and energy resolution in the self-biased (0 V applied bias) mode of operation. Self-biased operation is a highly sought after property for remotely operated detectors where carrying power supplies is a challenge, such as in space missions [35,36]. The average barrier height calculated using Equation (6) below was found to be 2.33 eV [37].

(6) $$\Phi_{B(C-V)}=V_{bi}+V_{n}$$

where, $V_{n}$ is the potential difference between the Fermi level and the conduction band minimum in the neutral region of the semiconductor. $V_{n}$ is expressed as

(7) $$V_{n}=k_{B}Tln\frac{N_{C}}{N_{D}}$$

where $N_{C}$ is the effective density of states in the conduction band of 4H-SiC and is taken to be 1.6 × 10^19^ cm^−3^, and $N_{D}$, the donor concentration, is taken to be equal to $N_{eff}$.

### 3.2. Radiation Detection

**Figure 3:**
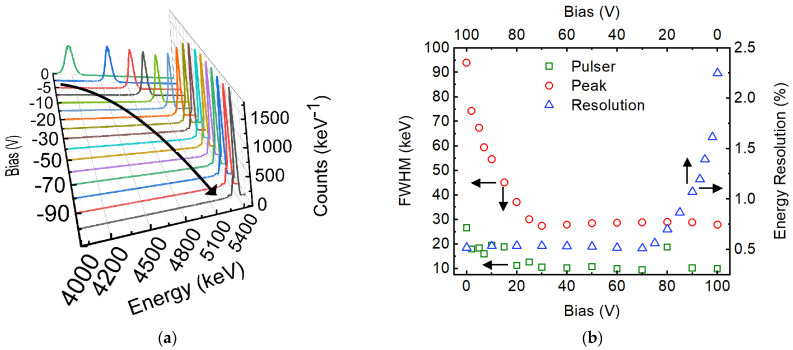
(**a**) Evolution of the PHS with increasing reverse bias obtained using a Pd/4H-SiC SBD. Separate colors have been used for different bias voltages. The curved arrow is a guide to the eye indicating the shift in peak position; (**b**) Variation of energy resolution expressed in terms of peak width (left *y*-axis) and percentage resolution (right *y*-axis). The arrows show the axes each plot is attributed to.

The junction properties observed in the Pd/4H-SiC SBD from the electrical measurements indicate high-resolution and high-efficient detection performance. Figure 3a shows the pulse height spectra obtained at different bias voltages starting from 0 V to −100 V with the detector illuminated by the alpha particle source. The self-biased detector showed a robust peak corresponding to the 5486-keV alpha particles. As is evident from the increase in the height of the peak corresponding to the 5486-keV alpha particles and its shift towards the higher energy channel in Figure 3a, the performance of the detector improved consistently with increase in reverse bias. The shift of the peak in the energy channel indicates an increase in charge collection efficiency, and the increase in peak height indicates a narrower peak (high resolution) for a given data acquisition period. The data acquisition time for each spectrum was set to 300 secs. Figure 3b shows the variation of detector energy resolution as a function of reverse bias along with that of the pulser peak width. The pulser peak width, recorded along with the alpha PHS, is a measure of the electronic noise in the detector system, which also includes the noise due to the detector leakage current and capacitance. The percentage energy resolution has also been plotted in the same graph. The energy resolution could be seen to improve (decrease in FWHM) drastically until a reverse bias of −30 V. The improvement is primarily due to the lowering of the detector capacitance, which controls both the white series and pink noise. The lowering of the electronic noise is evident from the decrease in the pulser peak FWHM. Beyond −30 V, the change in the detector capacitance almost saturated, leading to the saturation of the detector resolution as well. The electronic noise (pulser width) was seen to be far below the detector peak width, even at the optimized detector bias.

**Figure 4:**
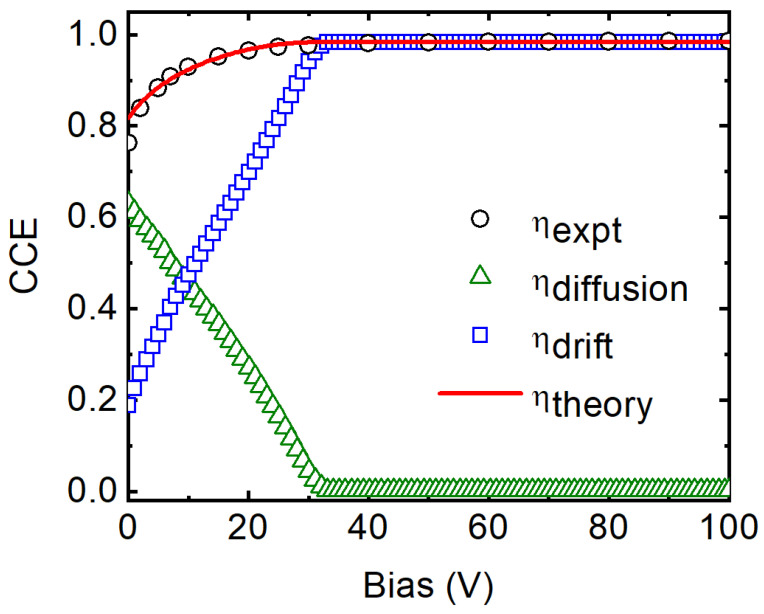
Variation of experimentally determined (open circle) CCE as a function of detector bias. The contribution of the diffusion (open triangle) and drift (open squares) motion of charge carriers to the overall CCE has been derived from a drift-diffusion model and plotted. The overall fit is shown by the solid line.

Figure 4 shows the variation of the CCE determined experimentally ($\eta_{expt}$) as a function of the reverse bias. As expected, the CCE improved with reverse bias up to −30 V due to the increase in drift velocity. At lower bias, and with the depletion width being narrow, most of the charge pairs are created beyond the depletion width i.e., in the neutral region. Since the electric field is almost absent within the neutral region, the only charges that are collected by the detector are the holes that reach the edge of the depletion width. With increasing bias, the depletion width increases, and the number of electrons created within the depletion width along the path of the alpha particles and the number of holes reaching the edge of the depletion width both increase, thereby increasing the charge collection efficiency significantly. The contribution from drift and diffusion of the charge carriers separately to the overall charge collection efficiency can be determined by fitting the variation of $\eta_{expt}$ as a function of the reverse bias to a drift-diffusion model, given by Equation (8) below [38].

(8) $$\eta_{theory}=\frac{1}{E_{p}}\int_{0}^{d}\left(\frac{dE}{dx}\right)dx+\frac{1}{E_{p}}\int_{d}^{x_{r}}\left[\left(\frac{dE}{dx}\right)\times exp\left\{-\frac{\left(x-d\right)}{L_{d}}\right\}\right]dx,\;=\eta_{drift}+\eta_{diffusion.}$$

In the above equation, d is the depletion width at a set bias, $x_{r\;}$is the penetration depth of the incident alpha particles in 4H-SiC, dE/dx is the electronic stopping power of the alpha particles calculated using SRIM 2013 [39], and $L_{d}$ is the minority carrier (hole) diffusion length. It can be observed from the plot in Figure 4 that $\eta_{diffusion}$ dominates over $\eta_{drift}$ until a reverse bias of −30 V, where the depletion width becomes comparable to the penetration depth of the alpha particles and the CCE is dominated by the drifting of charge carriers within the depletion width. Hence, above a reverse bias of −30 V, the $\eta_{drift}$ values approached the $\eta_{expt}$ values. From the fitting, the $L_{d}$ was calculated to be 30.8 μm, which is much longer than the 18.6 μm calculated in the benchmark Ni/4H-SiC SBD.

**Figure 5:**
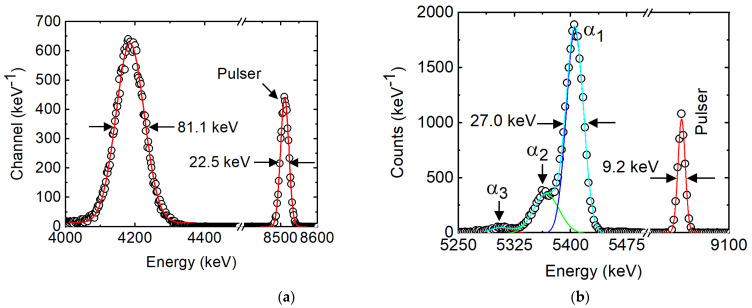
Pulse height spectra obtained using a Pd/4H-SiC SBD when exposed to a 0.9 μCi ^241^Am source emitting primarily 5486 keV alpha particles under self-bias (**a**); optimum bias (**b**). The solid lines are Gaussian peak fitting.

As mentioned earlier, in the present detector a robust alpha peak at zero applied bias has been obtained. The CCE in the self-biased mode was calculated to be ~76%, which is very close to that reported (≈82%) for 4H-SiC detectors with the highest CCE in self-biased mode [40]. The energy resolution of the detector in the self-biased mode was calculated to be 1.9% (81 keV) for the 5486 keV alpha particles, which is high enough for spectroscopic purposes. Such high CCE and energy resolution in the self-biased mode is due to the long hole diffusion length and the high built-in potential obtained for the Pd/4H-SiC SBD. The CCE and the energy resolution reached 98% and 0.49% (27 keV), respectively, for 5486 keV alpha particles at the optimized bias voltage of −50 V. The detailed pulse height spectra obtained in the self-biased mode and under the optimized biasing conditions are shown in Figure 5a,b, respectively. Table 1 summarizes the junction and the detector properties obtained for the present detectors.

### 3.3. Defect Characterization

**Figure 6:**
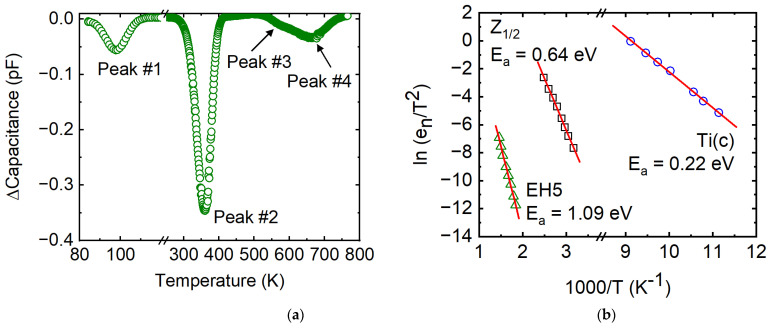
(**a**) A typical DLTS spectra obtained from the Pd/4H-SiC SBD; (**b**) Arrhenius plots corresponding to Peak #1, Peak #2, and Peak#4.

A typical DLTS spectrum obtained for the Pd/4H-SiC SBD is plotted in Figure 6a, which shows three well-resolved and one partially resolved peaks in the temperature scan range 80–790 K. The peaks in a DLTS scan form due to the resonant emission rate of trapped electrons from a trap center at a particular rate window setting [41]. The capacitance transients $C\left(t\right)$ due to the thermal emission of charge carriers from a trapping center are given by

(9) $$C\left(t\right)=C_{0}+\sum_{i}∆Cexp(-e_{n,i}t),$$

where ∆C is the capacitance change at time t= 0 due to charge trapping by the trap center indexed i, $C_{0}$ is the background capacitance at 0 V. The emission rate $e_{n,i}$ of the ith trap center is described by the Equation (10) below.

(10) $$e_{n,i}=\sigma_{n}\langle v_{th}\rangle N_{c}e^{\left(-\frac{E_{c}-E_{t}}{k_{B}T}\right)},$$

where $\langle v_{th}\rangle$ is the mean thermal velocity of charge carriers, $\sigma_{n}$ is the capture cross-section, and $E_{c}-E_{t}=E_{a}$ is the activation energy (considering electron trap centers) calculated as the energy level of the trap center $E_{t}$ measured relative to the conduction band minimum $E_{c}$. For an initial delay $t_{d}$ and a time $t_{2}$ after a rate window τ, the DLTS signal *S_i_*(*T*) is expressed as,

(11) $$C_{i}\left(t_{d}\right)-C_{i}(t_{2})=∆CS_{i}(T).$$

From the emission rate corresponding to the peaks, the Arrhenius plots can be generated, following Equation (10), to calculate the activation energies and capture cross sections of the trap centers. The trap concentration $N_{t}$ is calculated using Equation (12) below [41].

(12) $$\frac{N_{t}}{N_{eff}}=\frac{2∆C}{C(V_{p})}.$$

Figure 6b shows the Arrhenius plots corresponding to the three fully resolved peaks observed in the DLTS spectra. The partially resolved peak could not be analyzed due to the high uncertainty in the peak position. The defect parameters calculated from the Arrhenius plots and Equation (12) have been provided in Table 2. Peak #1 situated 0.22 eV below the conduction band minimum is well-studied and has been identified with the transition from the +3-charge state to +2 of substitutional titanium in the cubic lattice site and is labelled as Ti(c) [42]. The Ti impurity appears from the Ti crucible used in the growth process and is known to appear in as-grown SiC [43,44]. Peak #2 located 0.64 eV below the conduction band minimum corresponds to the Z_1/2_ center which has been identified as carbon vacancies from annealing studies using DLTS and electron paramagnetic resonance (EPR) [16,25,45,46]. Peak #4 is located at 1.09 eV below the conduction band minimum and resembles the EH5 trap center. EH5 trap center has been assigned to a carbon-related antisite–vacancy (CAV) pair in 4H-SiC [47,48].

## 4. Discussion

The energy resolution of 4H-SiC Schottky barrier detectors primarily depends on three factors: semiconductor material properties, Schottky junction properties, and the defect parameters. While the material properties such as effective doping concentration determines the junction capacitance and hence controls the white series and pink noises, junction parameters such as barrier height control the leakage current which in turn defines the white parallel noise in the spectrometer. White series, white parallel, and the pink noise are the three primary noise factors that define the overall electronic noise of a radiation spectrometer. Despite having vital device parameters such as barrier height, leakage current, built-in potential, hole diffusion length, and self-biased performance at par or even better than the Ni/4H-SiC benchmark SBD, the energy resolution of the Pd/4H-SiC SBD was found to be slightly lower when compared to the 0.29% observed for 5486 keV alpha particles using the benchmark detector. The electronic noise was observed to be much below the detector peak width at the optimized spectrometer setting, signifying that the detector performance is not limited by the electronic noise of the spectrometer. The above facts indicated that the detector performance is limited by the charge trapping in the lattice defects.

Capacitance mode deep level transient spectroscopy showed the presence of three trapping centers. While the Ti(c) centers are shallow impurity centers, the Z_1/2_ centers are very strong trapping centers and have been dubbed as the “lifetime killer” defect. The EH_6/7_ normally observed in our 4H-SiC epilayers has not been seen in these detectors, and instead a defect level with activation energy identical to EH5 center has been observed. Although EH5 is a deep defect level, the corresponding capture cross-section has been found to be on the order of 10^−17^ cm^2^, which is two orders of magnitude lower than the typical capture cross-sections of deep level defects, suggesting weak electron trapping in these defects. This further indicates that the Z_1/2_ center is the defining trapping center in the Pd/4H-SiC detector as far as the effect of charge carrier trapping is concerned. The benchmark detector reports a concentration of ≈10^11^ cm^−3^ for the Z_1/2_ center, which is one order of magnitude lower than that observed in the present detector [22]. Hence, the observed difference in the energy resolution of the two detectors is attributed to the varied concentration of the Z_1/2_ center.

In the self-biased mode, the Pd/4H-SiC SBD showed a much higher charge collection efficiency of 76% and an energy resolution of 1.9%; this is compared to the 57% charge collection efficiency and energy resolution of 4.3% observed in the benchmark Ni/4H-SiC SBD when exposed to 5486 keV alpha particles. The superior performance has been attributed to a longer hole diffusion length and a strong built-in potential.

Surface defects also play an important role in defining the detector performance by controlling the surface recombination velocity and surface leakage current. The effect of surface defects and surface currents in Schottky diodes may be greatly reduced by using surface passivation techniques and guard ring structures, respectively. The Pd/4H-SiC SBD and the benchmark detector are both fabricated under the same conditions where no surface passivation technique or guard ring structure has been used. Hence, the role of surface properties in the observed behavioral difference between the two detectors is thought to be minimal.

As a future plan, the authors intend to study the tolerance of the Pd/4H-SiC detectors in harsh environment conditions such as in high background radiation and high temperature environments. The authors also plan to study the effect of interfacial defects on the device performance and compare them to those observed in the benchmark detector.

## 5. Conclusions

Schottky barrier detectors have been fabricated by depositing 20 nm thick palladium metal films to assess their performance as radiation detectors. The Pd/4H-SiC SBD showed excellent rectification behavior, with a reverse current density lower than that observed in benchmark Ni/4H-SiC SBD at high operating voltages. The fabricated Pd/4H-SiC SBD showed a high barrier height of 1.5 eV and an ideality factor of 1.1. The ideality factor indicated spatial inhomogeneity in barrier height, which implies the presence of low barrier patches. Indeed, the average barrier height calculated from the *C-V* measurements was found to be 2.33 eV. The *C-V* measurements also showed a high built-in potential of 2.03 V. A high hole diffusion length of ≈30.8 μm was calculated using a drift-diffusion model applied to the alpha pulse height spectroscopy. The high built-in potential and the long hole diffusion length are sufficient to ensure spectroscopic grade radiation detection even in self-biased mode. In fact, the Pd/4H-SiC SBD showed an energy resolution of 1.9% and charge collection efficiency of 76% in self-biased mode which are superior to those reported for the benchmark detector. Despite having junction properties at par with the benchmark detector, the Pd/4H-SiC SBD showed a slightly lower energy resolution of 0.49% at optimized bias, compared to that of 0.29% reported for the benchmark detector for 5486 keV alpha particles. Capacitance mode DLTS measurements showed the presence of two deep levels and one shallow level trap center. The shallow level has been identified as the titanium impurity center whereas the deep levels have been identified as the Z_1/2_ center corresponding to carbon vacancies and the EH5 center corresponding to the carbon-antisite vacancy pair. The resolution of the fabricated Pd/4H-SiC SBD was found to be limited by the higher concentration of the Z_1/2_ center, which in the benchmark detector was found to be one order of magnitude less. It has been concluded that Pd/4H-SiC Schottky barrier detector has the potential to exhibit superior performance compared to conventional Ni/4H-SiC SBDs provided the effect of the Z_1/2_ centers are mitigated.

## Figures and Tables

**Table 1:** Detector parameters calculated from the electrical and PHS measurements.

Leakage Current Density at −200 V (nA/cm^2^)	SBH (eV)	*n*	*V_bi_* (V)	*N_eff_* (cm^−3^)	*L_d_* (μm)	Optimum Energy Resolution for 5486 keV Alpha Particles (keV)
0.4	1.5	1.1	2.03	1.63 × 10^14^	30.8	27 (0.5%)

**Table 2:** Defect parameters calculated from the DLTS measurements.

Trap ID	*E_a_* = *E_C_* − *E_T_* (eV)	*N_T_* (cm^−3^)	*σ* (cm^−2^)
Ti(c)	0.22	5.34 × 10^11^	2.15 × 10^−12^
Z_1/2_	0.64	3.55 × 10^12^	1.20 × 10^−15^
EH5	1.09	1.98 × 10^11^	1.66 × 10^−17^

## Data Availability

The data presented in this study are available on request from the corresponding author.
